# Transcriptome Analysis of Large to Giant Congenital Melanocytic Nevus Reveals Cell Cycle Arrest and Immune Evasion: Identifying Potential Targets for Treatment

**DOI:** 10.1155/2021/8512200

**Published:** 2021-12-06

**Authors:** Boxuan Wei, Jieyu Gu, Ran Duan, Bowen Gao, Min Wu, Shengliang Zhou, Xiaolu Huang, Feng Xie

**Affiliations:** Department of Plastic and Reconstructive Surgery, Shanghai Ninth People's Hospital, Medical College of Shanghai Jiaotong University, Shanghai, China

## Abstract

Large to giant congenital melanocytic nevus (lgCMN) is a benign cutaneous tumor that develops during embryogenesis. A large number of lgCMN patients are ineligible for surgical treatment; hence, there is an urgent need to develop pharmacological treatments. Clinically, tumorigenesis and progression essentially halt after birth, resulting in the homeostasis of growth arrest and survival. Numerous studies have employed whole-genome or whole-exome sequencing to clarify the etiology of lgCMN; however, transcriptome sequencing of lgCMN is still lacking. Through comprehensive transcriptome analysis, this study elucidated the ongoing regulation and homeostasis of lgCMN and identified potential targets for treatment. Transcriptome sequencing, identification of differentially expressed genes and hub genes, protein–protein network construction, functional enrichment, pathway analysis, and gene annotations were performed in this study. Immunohistochemistry, real-time quantitative PCR, immunocytofluorescence, and cell cycle assays were employed for further validation. The results revealed several intriguing phenomena in lgCMN, including *P16*-induced cell cycle arrest, antiapoptotic activity, and immune evasion caused by malfunction of tumor antigen processing. The arrested cell cycle in lgCMN is consistent with its phenotype and rare malignant transformation. Antiapoptotic activity and immune evasion might explain how such heterogeneous cells have avoided elimination. Major histocompatibility complex (MHC) class I-mediated tumor antigen processing was the hub pathway that was significantly downregulated in lgCMN, and *ITCH*, *FBXW7*, *HECW2*, and *WWP1* were identified as candidate hub genes. In conclusion, our research provides new perspectives for immunotherapy and targeted therapy.

## 1. Introduction

Congenital melanocytic nevus (CMN) is a neurocutaneous benign tumor that results from abnormal proliferation and migration of melanocytes during embryogenesis. Based on the measurement of the largest projected diameter of nevus at adulthood, CMN is classified into small (<1.5 cm), medium (1.5–20 cm), large (20–40 cm), and giant (>40 cm) types [1]. The large to giant CMN (lgCMN) is more difficult to manage and has a heavier psychological burden and a higher malignant transformation rate than small to medium CMN [2]. The overall risk of developing melanoma in lgCMN patients is approximately 2% [3]. Several lgCMN cases also demonstrate complications, including neurocutaneous melanosis, neural dysplasia, neurofibromatosis, lipoma, and vitiligo [4–6]. These characteristics have gradually brought lgCMN to the forefront of pediatrists' and dermatologists' attention.

Surgical resection and tissue expansion remain the mainstay of lgCMN treatment [7, 8]. However, patients often endure great suffering and have a risk of developing certain complications during the long surgical treatment period [9]. Moreover, patients with extensive principle lesions are ineligible for surgical treatment. Hence, effective therapies should be explored and developed to improve the management of lgCMN. To enable research on potential treatment, we should shed light on the etiology, tumorigenesis, and homeostasis of lgCMN, in which genetics and bioinformatics play a crucial role.

Previous studies have reported common somatic mutations in *NRAS* and *BRAF* in CMN. *NRAS* mutations are identified in 50–80% of patients with lgCMN [10, 11], and *BRAF* mutations are frequently detected in small CMN [10]. Similarly, malignant melanomas frequently harbor *BRAF* and *NRAS* somatic mutations [12, 13]. However, unlike the significant proliferation and invasion of melanoma, lgCMN mostly demonstrates a steady state without growth and migration. Clinically, lgCMN mostly demonstrates arrested growth after birth, with no increase in the lesions, no trend of increasing satellite nevi, and a low malignant transformation rate. Without any treatment or interference, these lesions may survive and last a lifetime [14]. Both *in vivo* and *in vitro*, lgCMN cells are nontumorigenic with a finite lifespan [15] and are able to mediate growth and antiapoptosis processes to increase the survival potential [16]. These features suggest that lgCMN has developed homeostasis during growth arrest and survival. Growing numbers of whole-genome or whole-exome sequencing have been performed to elucidate the etiology and tumorigenesis of lgCMN; however, none of these studies have focused on the transcriptomics of lgCMN. The state of regulation and homeostasis existing in lgCMN remain unclear. To explore the biological processes occurring in lgCMN, there is an urgent need for improved transcriptome analysis.

In this study, based on the transcriptome analysis results, we propose intriguing phenomena that are seen with lgCMN: growth arrest and immune evasion. Functional enrichment was performed to determine the regulation between different pathways or processes. Finally, we unveiled the hub pathways and candidate genes that provide a framework for further research in targeted therapy and immunotherapy.

## 2. Materials and Methods

### 2.1. Clinical Specimens

We enrolled patients diagnosed with lgCMN at the Shanghai Ninth People's Hospital between 2019 and 2020. The projected adult size of the nevus was estimated to obtain an accurate diagnosis. All patients underwent surgical resection as treatment. We obtained 12 lgCMN samples and 4 lgCMN-adjacent normal skin samples for transcriptome analysis. Expression data of transcriptome is provided in Supplementary Materials 1, and the detailed information on the samples is presented in Table 1.

### 2.2. RNA Extraction

For all samples in Table 1, total RNA was isolated using the UNlQ-10 RNeasy Kit (Sangon, Shanghai, China). RNA integrity was checked before RNA sequencing using the Agilent 2100 Bioanalyzer (Agilent Technologies, Santa Clara, CA, USA).

### 2.3. RNA Sequencing

For cDNA construction, total RNA was extracted from the samples and processed using a TruSeq RNA Sample Preparation Kit (Illumina, Shanghai, China), and cDNA libraries were used to generate paired-end reads. Sequencing was performed on each library to generate 100 bp paired-end reads for transcriptome sequencing on an Illumina High-Seq 2000 platform (Illumina, San Diego, CA, USA) by a commercial service provider (Sangon).

### 2.4. Identification of Differentially Expressed Genes (DEGs)

The DEGs in lgCMN tissue samples were screened using the limma package in R [17]. The adjusted *P* value (*Q* value) and Benjamini–Hochberg false discovery rate were applied to balance the discovery of significant genes and to limit of false positives. Log | FC | >2 and *Q* value < 0.05 were considered significant in this study.

### 2.5. Protein–Protein Interaction (PPI) Network Construction and Module Analysis

The PPI network was constructed using STRING [18]. Cytoscape is a bioinformatics software used for visualizing molecular interaction networks [19]. The plug-in app, molecular complex detection (MCODE), of Cytoscape was used to identify the most densely connected module in the PPI network [20, 21]. The thresholds were as follows: MCODE scores > 5, max depth = 150, degree cut‐off = 2, node score cut‐off = 0.2, and *k*‐score = 2.

### 2.6. Gene Ontology and Pathway Enrichment Analyses

Gene Ontology (GO) and Kyoto Encyclopedia of Genes and Genomes (KEGG) pathway enrichment analyses were performed using the Database for Annotation, Visualization and Integrated Discovery (DAVID) [22, 23]. The hub pathways were identified using Reactome, a pathway database [24]. A *Q* value < 0.05 was considered significant.

### 2.7. Cell Culture and Cell Cycle Assays

Normal human melanocytes (NHM) were isolated from healthy skin tissues, and lgCMN cells were isolated from patients' tumor lesions, following previously described procedures [25, 26]. Both NHM and lgCMN cells were cultured in melanocyte medium (ScienCell, Carlsbad, CA, USA) according to the manufacturer's instructions. The human melanoma cell line A375 was purchased from American Type Culture Collection (Manassas, VA, USA) and grown in RPMI-1640 medium containing 10% fetal bovine serum (Gibco, San Diego, CA, USA) and 1% antibiotic antimycotic solution (Gibco) at 37°C and 5% CO_2_. For cell cycle assays, A375 cells, P2 NHM, and P2 lgCMN cells were prepared in phosphate-buffered saline (PBS) at a density of 5 × 10^5^ cells/mL. The cell suspensions were pipetted into 70% ethanol on ice and kept for two hours. After washing the cells with PBS, they were stained using Cell Cycle and Apoptosis Analysis Kit (Beyotime, Shanghai, China) for two hours. A flow cytometer, Beckman CytoflexS (Beckman Coulter, Brea, CA, USA), was used to evaluate the cell cycle.

### 2.8. Immunohistochemistry

Immunohistochemical staining of formalin-fixed, paraffin-embedded normal skin tissues, lgCMN tissues, and lgCMN-developed melanoma tissues was performed on a Leica BOND MAX Immunostainer (Leica Microsystems, Wetzlar, Germany). The sections were pretreated using heat-mediated antigen retrieval with citrate buffer (pH 6.0) for 30 min. The sections were then incubated with Ki67 (ab16667, Abcam, Cambridge, UK, 1 : 100), P16 (MAB-0673, Maxim Biotechnologies, Fujian, China, 1 : 100), SOX10 (GTX35085, GeneTex, Irvine, CA, USA, 1 : 100), CD3 (MAB-0740, Maxim Biotechnologies, 1 : 100), CD4 (RMA-0620, Maxim Biotechnologies, 1 : 100), CD8 (RMA-0514, Maxim Biotechnologies, 1 : 100), BCL2 (MAB-0711, Maxim Biotechnologies, 1 : 100), or caspase-3 (GTX110543, GeneTex, 1 : 100) for 1 h at 20–30°C and detected using an HRP-conjugated compact polymer system. AEC was used as the chromogen. The sections were then counterstained with hematoxylin.

### 2.9. Immunocytofluorescence

The cells were fixed with 4% paraformaldehyde for 10 min, permeabilized with 0.1% PBS-TritonX-100 for 5 min, and then blocked with 1% BSA/10% normal goat serum/0.3 M glycine in 0.1% PBS-Tween for 1 h. The cells were then incubated overnight at 4°C with anti-Ki67 antibody (ab16667, Abcam, 1 : 200)/anti-SOX10 antibody (GTX35085, GeneTex, 1 : 100) or anti-P16 antibody (ab81278, Abcam, 1 : 200)/anti-SOX10 antibody, followed by further incubation at 20–30°C for 1 h with goat anti-rabbit IgG H&L (FITC, ab6717, Abcam, 1 : 1000) and goat anti-mouse IgG H&L (Cy3 ®) preadsorbed (ab97035, Abcam, 1 : 500) secondary antibodies. Nuclear DNA was labeled blue with DAPI.

### 2.10. Real-Time Quantitative PCR

Real-time quantitative PCR was performed according to the MIQE guidelines [27]. After RNA extraction, cDNA was generated using high-capacity cDNA reverse transcription kit (AB Applied Biosystems, San Diego, CA, USA). Real-time quantitative PCR detection was conducted on a LightCycler® 480 Instrument II (Roche, Rotkreuz, Switzerland) using 2X SG Fast qPCR Master Mix (BBI Life Sciences, Shanghai, China) and specific primers. PCR data were analyzed using the 2^−*ΔΔ*Ct^ method. *GAPDH* is applied to normalize the data. The primers are listed in Supplementary Materials 2.

### 2.11. Statistical Analysis

Statistical analyses were performed using GraphPad Prism 8. Student's *t*-test was used for the analysis of differences between two groups, and the ANOVA was conducted for comparisons among the groups. Data are presented as the mean ± SD. A *P* value < 0.05 was considered to be statistically significant.

## 3. Results

### 3.1. Identification of DEGs in the lgCMN Transcriptome

**Figure 1:**
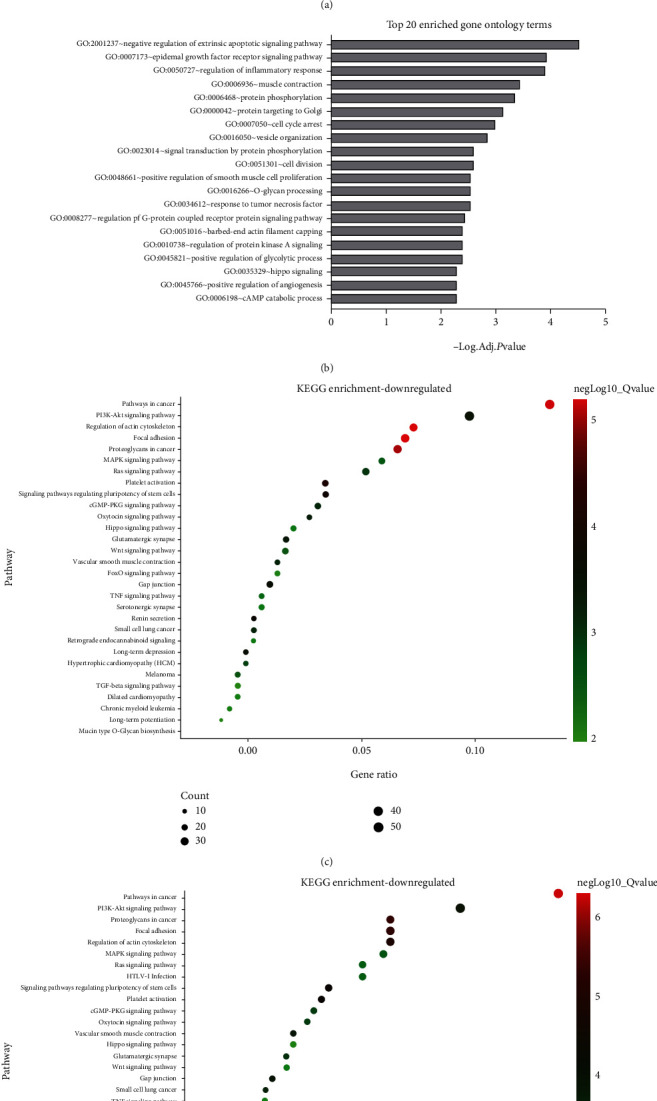
Functional enrichment of 1562 DEGs in lgCMN. (a) Volcano plot of DEGs. The red dots represent the upregulated genes, whereas the green dots represent the downregulated genes. The gray dots are genes without significant difference in expression. Line threshold: Log  | FC | >2 and adjusted *P* value < 0.05. (b) Gene Ontology enrichment of DEGs. (c and d) Top 30 significantly enriched pathways of downregulated and total DEGs demonstrated as bubble charts. The size of the bubble illustrates the gene count in each pathway, and the color symbolizes the enrichment degree. A *Q* value < 0.05 was considered statistically significant. DEGs: differentially expressed genes; GO: Gene Ontology; KEGG: Kyoto Encyclopedia of Genes and Genomes; *Q* value = adjusted *P* value.

The DEGs between lgCMN and lgCMN-adjacent normal skin samples were identified by analyzing the gene expression data. Using a threshold of Log | FC | >2 and adjusted *P* value < 0.05, 165 upregulated and 1397 downregulated genes were detected. This result is shown as a volcano plot (Figure 1(a)), and these DEGs are listed in Supplementary Materials 3. The identified DEGs mostly comprised downregulated genes suggesting that several biological processes and pathways may be suppressed in the lgCMN tissues. We believe that the downregulation of these genes and pathways may be the pivotal mechanisms responsible for the benign characteristics and survival of lgCMN. Therefore, Gene Ontology and KEGG pathway enrichment analyses of the DEGs were performed.

### 3.2. Gene Ontology Enrichment Showed Antiapoptotic Activity and Cell Cycle Arrest in lgCMN

Gene Ontology enrichment (Figure 1(b)) showed downregulation of apoptosis regulation, inflammatory response, and response to tumor necrosis factor (TNF). These changes may prevent apoptosis and protect lgCMN from programmed cell death. Moreover, cell cycle arrest and cell division, which regulate cell proliferation, were also enriched. These findings imply that lgCMN is in a state of cell cycle arrest and antiapoptosis.

To further investigate the mechanisms of maintaining growth arrest and survival in lgCMN, we performed KEGG pathway enrichment analysis of the upregulated genes, downregulated genes, and all DEGs. The results indicated that 90% (27/30) of the pathways enriched by all DEGs were also found to be enriched by the downregulated genes (Figures 1(c) and 1(d)). That implies that the downregulated genes practically enriched the pathways in all DEGs, and these were regarded as downregulated pathways in lgCMN.

### 3.3. Pathways Associated with Tumor Proliferation and Migration Were Downregulated in lgCMN

Pathways in tumorigenesis, including PI3K-AKT (*P* = 3.39*e* − 04), RAS (*P* = 0.0012), MAPK (*P* = 0.0015), melanoma (*P* = 0.0023), and WNT signaling pathways (*P* = 0.0073), were significantly downregulated (Figures 1(c) and 1(d)). Pathways involved in tumor migration were also inhibited, including focal adhesion (*P* = 9.72*e* − 06), proteoglycans in cancer (*P* = 5.00*e* − 06), and regulation of the actin cytoskeleton (*P* = 1.49*e* − 05). Several pathways that induce tumor proliferation, differentiation, and migration were significantly downregulated in lgCMN, which may explain how lgCMN retains its benign characteristics and growth arrest.

### 3.4. Immuno- and Apoptosis-Related Pathways Were Suppressed in lgCMN

Expression of TNF signaling pathway (*P* = 0.0061) and its downstream NF-*κ*B pathway (*P* = 0.0128) was also inhibited, causing a reduction in inflammatory factor levels and affecting the process of apoptosis and immune response. Moreover, caspase-3 (Log  | FC | = −2.80) and caspase-7 (Log | FC | = −1.56), the key effectors inducing apoptosis, levels were also downregulated.

### 3.5. PPI Network Construction and Key Module Detection

**Figure 2:**
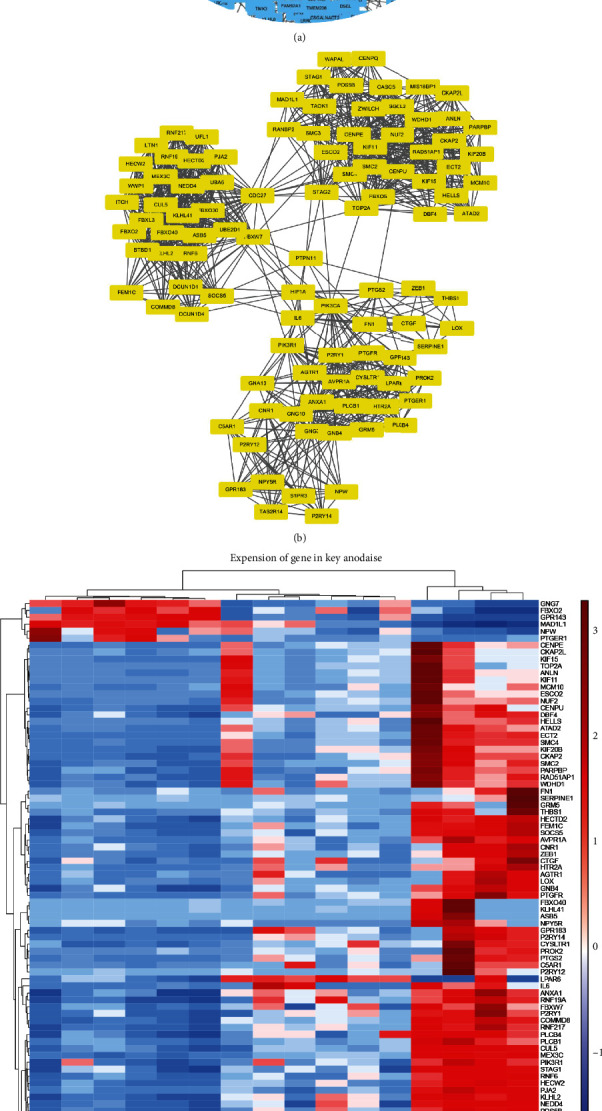
PPI network construction and key module detection. (a) PPI network of the DEGs created using Cytoscape. The genes of the key module are in yellow, whereas the other DEGs are in blue. (b) Key module identified using the MCODE plug-in of Cytoscape. The key module possesses the most frequent and closest interactions in the PPI network. (c) Heatmap and cluster analysis of key module genes. Red and blue depict relative gene expression levels from high to low. Heatmap scale: row; cluster method: ward; D2; row distance measure: Euclidean; row distance measure: Euclidean. PPI: protein–protein interaction; DEGs: differentially expressed genes; MCODE: molecular complex detection.

To clarify the reciprocal actions of the 1562 DEGs, a PPI network was constructed (Figure 2(a)). Subsequently, the cluster with the most frequent and closest gene interactions was screened using the plug-in component MCODE in Cytoscape, which is defined as the “key module” in this study [20, 21]. With the highest MCODE score of 21.64, the key module consisted of 6 upregulated and 95 downregulated genes, containing 1121 interacting edges (Figure 2(b)). Owing to their dense protein interactions, the genes of the key module are extremely likely to make significant contributions to lgCMN homeostasis (Figure 2(c)).

### 3.6. Identification of Hub Pathways

**Figure 3:**
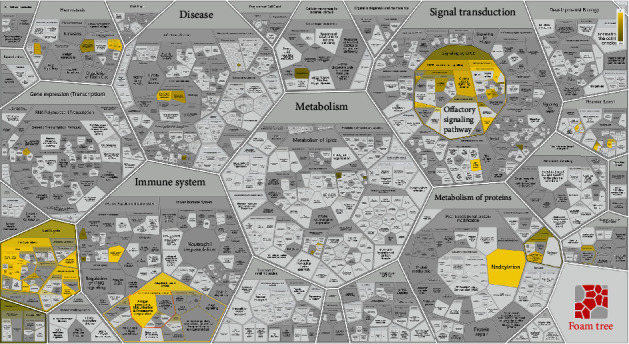
Reactome pathway enrichment of key module genes. The brighter the color, the more significant the pathway enrichment. The analyses were done using the Reactome pathway database. A *Q* value <0.05 was considered statistically significant.

Further identification of hub pathways and genes is necessary to identify potential targets for lgCMN treatment. Hence, we subsequently performed Reactome pathway enrichment analysis on the key module because as compared to KEGG, Reactome provides a detailed pathway enrichment of specific parts of biological processes and molecular functions, and thus, it is more suitable for key module analysis [24]. The results are shown as a foam tree (Figure 3). The most prominent sections were antigen processing and presentation of the adaptive immune system, cell cycle, and neddylation, and all these were enriched by downregulated genes.

### 3.7. *P16*-Induced Cell Cycle Arrest in lgCMN

**Figure 4:**
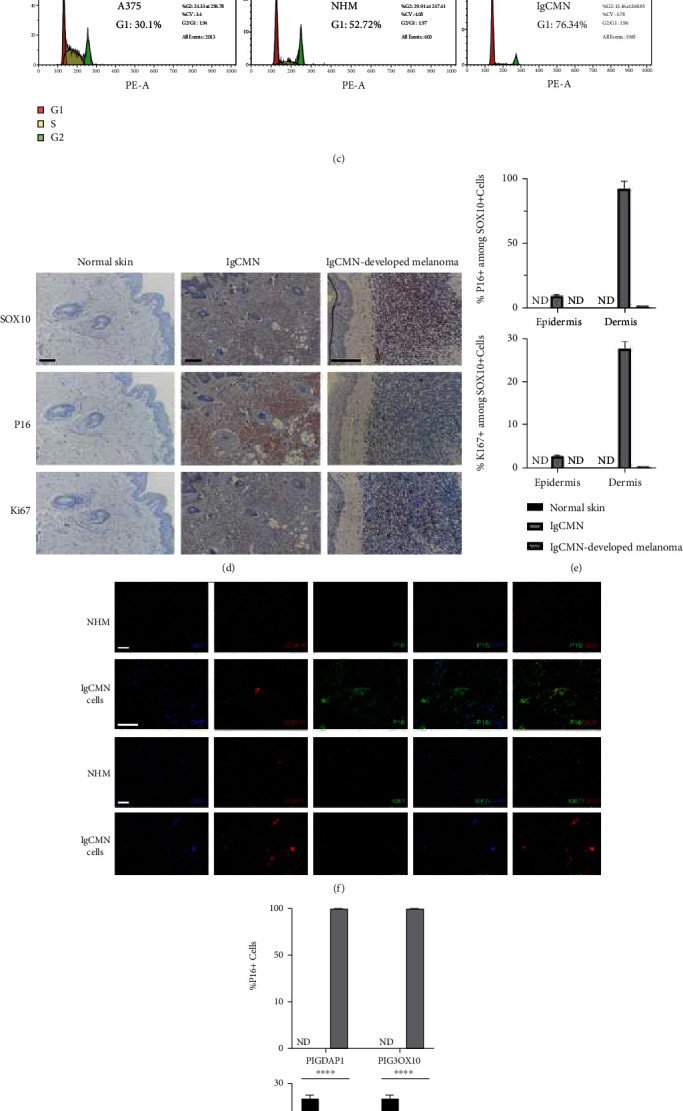
Cell cycle arrest and significantly upregulated *P16* expression in lgCMN. (a) Relative gene expression of cell cycle inhibitors in lgCMN and normal skin tissues. Red bars represent upregulation, and blue bars represent downregulation. (b) Morphology of A375, NHM, and lgCMN cells with different shapes and number of dendrites. (c) Cell cycle assays. P2 cells of each cell line were cultured for the experiment. (d) Immunohistochemical staining for SOX10 (upper panels), P16 (middle panels), and Ki67 (lower panels) of normal skin (*n* = 5; patient 2 + patient 4 + patient 5 + patient 9 + patient 10), lgCMN (*n* = 5; patient 2 + patient 4 + patient 5 + patient 9 + patient 10), and lgCMN-developed melanoma (*n* = 1; a supplementary lgCMN patient with malignant transformation at 4 years of age). SOX10, P16, and Ki67 were stained using the AEC chromogen. (e) Histograms show the number of P16+ or Ki67+ cells per SOX10+ cells in the epidermis and dermis. (f) Immunofluorescent staining of SOX10, P16, and Ki67 in P2 NHM (*n* = 3; patient 2 + patient 9 + patient 10) and P2 lgCMN cells (*n* = 3; 3 foreskin samples from different children). P16+ and Ki67+ cells were stained using FITC-conjugated antibodies, and SOX10+ cells were stained using Cy3-conjugated antibodies. (g) Histograms show the number of P16+ or Ki67+ cells per DAPI and SOX10+ cells. ^∗∗∗∗^*P* < 0.0001 represents differences between NHM and lgCMN in the *t*-test. Error bars represent the mean ± SD. lgCMN: large to giant congenital melanocytic nevus; NHM: normal human melanocytes; P2: passage 2; ND: not detected; SD: standard deviation. Scale bar = 50 *μ*m.

According to key module functional enrichment, cell cycle progression was inhibited, indicating that lgCMN is under cell cycle arrest. To explore the mechanisms underlying the induction of cell cycle arrest in lgCMN, we compared the expression of cell cycle inhibitors. Among them, we found that *P16* (*CDKN2A*) which plays crucial roles in growth homeostasis was significantly upregulated in lgCMN samples (Figure 4(a)). Further validation was performed to support this conclusion. Primary NHM and lgCMN cells were isolated from patients' tissues (Figure 4(b)). Cell cycle assays of A375, NHM, and lgCMN cells were performed. Compared to NHM and A375 cells, lgCMN cells demonstrated a very high proportion of cell cycle arrest (Figure 4(c)).

Moreover, we performed immunohistochemistry and immunocytofluorescence on lgCMN and normal samples and identified the expression of Ki67, P16, and SOX10. Paraffin sections for immunohistochemistry of normal skin tissues and lgCMN tissues were prepared from five different patients and one lgCMN-developed melanoma tissue from another patient. SOX10 staining was performed to determine the distribution of normal and abnormal melanocytes. In normal skin tissue, SOX10 nuclear staining was found only in the basal epidermal cells, whereas extensive SOX10-positive cells were found in the dermis of lgCMN and melanoma tissues (Figure 4(d)). Among these SOX10-positive cells, lgCMN showed much higher expression of P16 than that in melanoma and normal skin tissues; however, the proliferative marker Ki67 exhibited low expression in lgCMN tissues (Figure 4(e)). These results established that most lgCMN cells undergo cell cycle arrest, and P16 is the mainstay of this phenomenon. In the lgCMN-developed melanoma tissue, the greatly decreased expression of P16 recovered cell cycle progression and promoted malignant transformation. Primary lgCMN cells and normal melanocytes extracted from fresh specimens were used for immunocytofluorescence. Likewise, lgCMN cells demonstrated a much higher positive rate of P16 and a lower positive rate of Ki67 than normal melanocytes (Figures 4(f) and 4(g)). In summary, these experiments revealed the *P16*-induced growth arrest in lgCMN, which is considered an essential process for preserving its benign characteristics.

### 3.8. Immune Evasion in lgCMN

**Figure 5:**
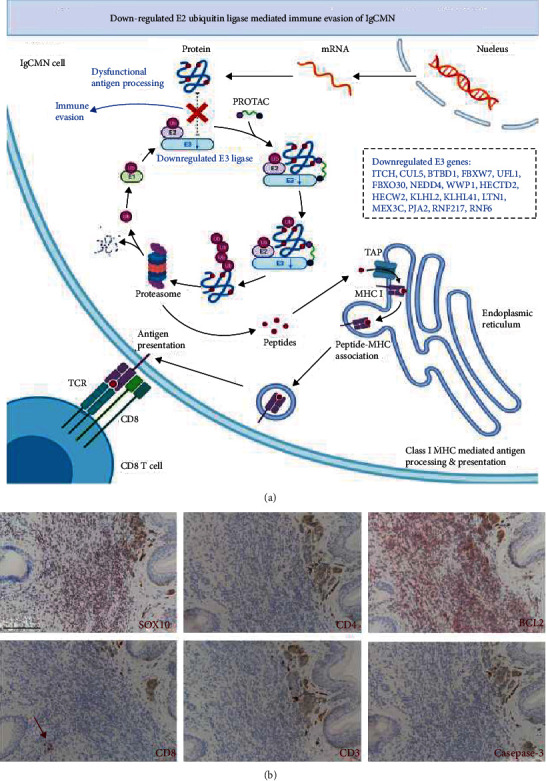
Immune evasion in lgCMN. (a) Flow chart of MHC class I-mediated antigen processing and presentation. The abnormal parts are presented in blue fonts. The downregulated E3 ligase gene leads to the dysfunction of antigen processing in lgCMN, inducing immune evasion. This figure was created using BioRender. (b) Deficiency of tumor-infiltrating lymphocytes in lgCMN tissues. Immunohistochemical staining for SOX10 (labeling lgCMN cells), CD3 (labeling mature T lymphocytes), CD4 (labeling helper T lymphocytes), and CD8 (labeling cytotoxic T lymphocytes) in lgCMN tissues (*n* = 5; patient 2 + patient 4 + patient 5 + patient 9 + a lgCMN patient with halo phenomenon) was stained using the AEC chromogen to detect tumor-infiltrating lymphocytes. BCL2 (antiapoptosis related) and caspase-3 (apoptosis related) were stained using the AEC chromogen to investigate antiapoptotic activity. The red arrow points at a few CD8^+^ T cells far away from SOX10+ lgCMN cells.

Based on the functional enrichment of key modules (Figure 3), primary consideration was given to the most significant pathway, namely, antigen processing: ubiquitination and proteasome degradation (*P* = 1.11*e* − 16). Among the three components (MHC class I, MHC class II, and complex) of the pathway, MHC I-mediated antigen processing was responsible for enrichment, and it was the initiator of tumor antigen recognition and CD8^+^ T cell activation [28]. The root of the pathway malfunction was harbored in E3 ubiquitin ligase, whose coding genes were widely downregulated in lgCMN (Figure 5(a)). Deficiency in E3 ubiquitin ligase can paralyze tumor antigen processing in lgCMN, eventually causing immune evasion. The downregulated E3 coding genes from the key module are listed and colored blue in Figure 5(a), and real-time quantitative PCR, which was used to detect the mRNA expression of these genes, showed significant downregulation in lgCMN (Fig. S1a). Additionally, we performed immunohistochemical staining for SOX10, CD3, CD4, CD8, BCL2, and caspase-3 in lgCMN tissues to detect the tumor-infiltrating lymphocytes (TILs) and antiapoptotic phenomenon. TILs were deficient in lgCMN, whereas extensive expression of antiapoptotic marker BCL2 was discovered (Figure 5(b)). These findings revealed immune evasion in lgCMN, and its dysfunctional antigen processing and presenting for T cell activation and mobilization may be a crucial promoter.

### 3.9. Candidate Hub Gene Identification

Next, gene annotations of the downregulated E3 coding genes were conducted to probe their potential pathogenesis. We considered *ITCH*, *FBXW7*, *HECW2*, and *WWP1* as candidate hub genes among the E3 ubiquitin ligases that participate in antigen processing based on gene annotations and reported studies. *ITCH* is involved in the regulation of many aspects of immune responses, including lymphoid cell differentiation, T cell activation, and the strength of the T cell receptor signal [29]. The downregulation of *ITCH* greatly influences the antigen recognition and activation of the adaptive immune system, thus leading to the inhibition of tumor immune response [30]. Another hub gene we should pay attention to is *FBXW7*. Dysfunctional *FBXW7* is responsible for defects in antigen peptide formation and presentation of tumor development and malignancies [31]. Additionally, *FBXW7* loss promotes resistance to anti-PD-1 therapy in melanoma, and the reactivation of *FBXW7* could improve the response to PD-1 blockade [32]. *HECW2* and *WWP1* were also considered hub genes because of their participation in neural development during the embryonic period. Melanocytes and nevus cells are derived from neural crest cells after a series of differentiation and migration processes. Remarkably, *HECW2* plays a vital role in the proliferation, migration, and differentiation of neural crest cells, which may be involved in lgCMN pathogenesis [33]. *WWP1* is indispensable for the proper polarization of developing neurons, and its deregulation may cause abnormal neuronal morphology and dysfunction [34]. In summary, genes inducing ubiquitination-mediated antigen processing were inhibited in lgCMN, and among these genes; we proposed *ITCH*, *FBXW7*, *HECW2*, and *WWP1* as candidate hub genes because of their function in immune responses and melanocyte development.

### 3.10. Transcriptional Levels of Candidate Hub Genes Were Verified Using the GEO Database

Gene expression data of the 12 lgCMN samples were downloaded from the GSE8525 dataset [35]. The results showed that *ITCH*, *FBXW7*, *HECW2*, and *WWP1* were also downregulated (Fig. S1b). As the most crucial effector of apoptosis, caspase-3 was also downregulated in these samples. Lastly, *P16* (*CDKN2A*), the key cell cycle inhibitor, was upregulated in both our samples and the GEO samples, and inhibitory downstream *CDK4* and *CDK6* activity was downregulated.

## 4. Discussion

Clinically, we observed that lgCMN generally demonstrates a state of growth arrest and survival after birth. Therefore, a series of regulatory mechanisms may flourish after birth to prevent the malignant transformation of lgCMN. Although lgCMN does not progress, the abnormal nevus cells are not cleared by the immune system and survive in the tissues, and the reasons for this remain unknown.

According to the results, arrested proliferation and immune evasion are the two main aspects that should be intensively explored. The arrested proliferation or migration of lgCMN cells is consistent with its phenotype and rare malignant transformation, and immune evasion explains how such heterogeneous cells have avoided elimination.

Attention should be paid to cell cycle arrest as it was discovered in both the DEGs and key module analyses Among the cyclin-dependent kinase inhibitors, *P16* expression was considerably upregulated in lgCMN, whereas its inhibitory downstream *CDK6*, *RB1*, and E2F transcription factor levels were downregulated (Supplementary Materials 2). Previous research has reported a *P16*-induced senescence-like cell cycle arrest of acquired nevus that prevented it from developing into melanoma [36]. By contrast, melanoma frequently harbors *P16* deletion or its downregulation, inducing tumor malignancy and metastasis [37]. Stefanaki et al. [38] investigated the expression of G1 cell cycle regulators in lgCMN and melanoma using immunohistochemistry. The results showed high expression of P16 but rare expression of the other indexes in lgCMN. Conversely, proliferation indexes Ki67, cyclin D1, and Rb levels were upregulated in melanoma, whereas P16 expression was notably decreased. Likewise, in our study, immunohistochemistry and immunocytofluorescence demonstrated high expression of P16 but low expression of Ki67, and the cell cycle assays indicated prominent cell cycle arrest in lgCMN cells. These results revealed a *P16*-induced homeostasis of growth arrest in lgCMN, which plays a crucial role in preventing malignant transformation. In the case of the extensive expression of *P16* in lgCMN, further research on targeting *P16* and *P16*-induced senescence-like homeostasis in lgCMN cells will be enlightening.

Antiapoptotic activity and immune suppression were the other interesting biological processes occurring in lgCMN, mainly through the downregulated TNF signaling and antigen processing pathways. TNF kills tumors directly and promotes the differentiation of T and B cells [39, 40]. Furthermore, the activated TNF pathway eventually induces apoptosis through caspase-3 (Log  | FC | = −2.80) [39]. These functional characteristics suggest that the downregulated TNF signaling pathways may promote antiapoptosis and immune suppression in lgCMN.

Finally, the role of the most significantly enriched pathway in our study, MHC class I-mediated antigen processing, should be highlighted (Figure 5). Tumor antigens are ubiquitinated and labeled in the cytoplasm of tumor cells before being hydrolyzed by the proteasome and processed into appropriate peptides [41]. Only specific antigen peptides are suitable for binding MHC I and are eventually transported to the cell membrane for presentation to CD8^+^ T cells. By recognizing the antigen peptides, CD8^+^ T cells eliminate tumor cells and subsequently mobilize the adaptive immune system [42]. As the initial step of antigen processing, ubiquitination requires the participation of E1 ubiquitin-activating enzyme, E2 ubiquitin-conjugating enzyme, and E3 ubiquitin ligase [43]. However, numerous components of E3 ubiquitin ligase complex were significantly downregulated in lgCMN, leading to malfunction of antigen ubiquitination. Tumor antigens cannot be ubiquitinated or linked to the proteasome. Without appropriate peptides, the presentation of CD8^+^ T cells is blocked, resulting in immune evasion of lgCMN [41–43]. Among the downregulated genes of E3 ubiquitin ligase, *ITCH*, *FBXW7*, *HECW2*, and *WWP1* were considered candidate hub genes in lgCMN according to gene annotations. To the best of our knowledge, immune-related studies in lgCMN have not yet been reported, and our results may inspire research on immunotherapy for lgCMN.

## 5. Conclusion

In this study, we provide the first comprehensive transcriptome analysis of lgCMN. We revealed intriguing phenomena, including P16-induced growth arrest, antiapoptotic activity, and *ITCH*-, *FBXW7*-, *HECW2*-, and *WWP1*-induced immune evasion in lgCMN. These discoveries may provide new perspectives for targeted therapy and immunotherapy.

## Figures and Tables

**Table 1:** Clinical characteristics of samples for transcriptome analysis.

Samples	Patient number	Sex	Age at biopsy date	Tissue site or 6B classification	PAS (cm)	CMN type	Satellites	New satellites formed after birth	Abnormal enlargement of the lesions after birth
Tumor 1	1	M	7 years	Scalp	20-30 cm	Large L1	20-50	No	No
Tumor 2	2	M	4 years	Back and breast	40-60 cm	Giant G1	No satellites	No	No
Tumor 3	3	M	4 years	Face and scalp	30-40 cm	Large L2	<20	No	No
Tumor 4	4	F	7 years	Bathing trunk	40-60 cm	Giant G1	20-50	No	No
Tumor 5				Thigh satellites	15-20 cm				
Tumor 6	5	M	8 years	Back	30-40 cm	Large L2	<20	No	No
Tumor 7				Back satellites	10-15 cm				
	6	M	1 years	Bolero and breast	>60 cm	Giant G2	>50	No	No
Tumor 8				Thigh satellites	5-10 cm				
Tumor 9	7	F	12 years	Face and neck	20-30 cm	Large L1	No satellites	No	No
Tumor 10	8	F	12 years	Arm	20-30 cm	Large L1	No satellites	No	No
Tumor 11	9	F	5 years	Bolero	40-60 cm	Giant G1	20-50	No	No
Tumor 12	10	F	20 years	Face and scalp	30-40 cm	Large L2	20-50	No	No
Normal 1	1	M	7 years	Adjacent normal skin	NA	NA	NA	NA	NA
Normal 2	2	M	4 years	Adjacent normal skin	NA	NA	NA	NA	NA
Normal 3	3	M	4 years	Adjacent normal skin	NA	NA	NA	NA	NA
Normal 4	4	M	7 years	Adjacent normal skin	NA	NA	NA	NA	NA

Patient 6 only underwent satellite excision because of age limitation. M: male; F: female; PAS: projected adult size; CMN: congenital melanocytic nevi; NA: not applicable.

## Data Availability

Gene expression data generated during RNA-seq are in Supplementary Materials 1. The other data that support the findings of this study can be made available by the corresponding author (FX) upon reasonable request.

## Abbreviations

BRAF: B-Raf proto-oncogene, serine/threonine kinase
CDKN2A: Cyclin-dependent kinase inhibitor 2A
DEGs: Differentially expressed genes
FBXW7: F-box and WD repeat domain-containing 7
HECW2: HECT, C2, and WW domain-containing E3 ubiquitin-protein ligase 2
lgCMN: Large to giant congenital melanocytic nevus
MHC: Major histocompatibility complex
NHM: Normal human melanocytes
NRAS: Neuroblastoma RAS viral oncogene
ITCH: Itchy E3 ubiquitin-protein ligase
SOX10: SRY-box transcription factor 10
TNF: Tumor necrosis factor
TILs: Tumor-infiltrating lymphocytes
WWP1: WW domain-containing E3 ubiquitin-protein ligase 1.

## Supplementary Materials

Supplementary Materials Figure S1: validation of candidate hub gene expression. Supplementary Materials 1: gene expression matrix. Supplementary Materials 2: PCR primers. Supplementary Materials 3: differentially expressed genes in lgCMN. Supplementary Materials 1, 2, and 3 may be downloaded from Mendeley datasets (doi: 10.17632/rvhfj2cc5k.1).
