# Supplementary material for: Transcriptome Analysis of Large to Giant Congenital Melanocytic Nevus Reveals Cell Cycle Arrest and Immune Evasion: Identifying Potential Targets for Treatment
Source: J Immunol Res. 2021 Dec 6;2021:8512200. doi: 10.1155/2021/8512200 (PMC8668353; doi:10.1155/2021/8512200)
Supplement: Supplementary Materials — Figure S1: validation of candidate hub gene expression. Supplementary Materials 1: gene expression matrix. Supplementary Materials 2: PCR primers. Supplementary Materials 3: differentially expressed genes in lgCMN. Supplementary Materials 1, 2, and 3 may be downloaded from Mendeley datasets (doi: 10.17632/rvhfj2cc5k.1). [file 8512200.f1.docx]

**Figure S1. Validation of Candidate hub gene expression.** (a) Relative mRNA expression between adjacent normal skin and lgCMN tissues was identified by Real-time quantitative PCR. The housekeeping gene is *GADPH*. Target genes are *ITCH*, *FBXW7*, *HECW2*, *WWP1*, *CUL5*, *BTBD1*, *UFL1*, *FBXO30*, *NEDD4*, *HECTD2*, *KLHL2*, *KLHL41*, *LTN1*, *MEX3C*, *PJA2*, *RNF217* (antigen processing-related), caspase-3 (apoptosis-related), *P16*, *CDK4*, *CDK6* (cell-cycle-related). (b) A heatmap demonstrates the expression of candidate hub genes. Twelve lgCMN samples from GEO database are labeled as T1 to T12, while four adjacent normal samples from our research were labeled as N1 to N4. The down-regulation and up-regulation of the genes were consistent with our previous results. *P<0.05, **P<0.01, ***P<0.001, ****P<0.0001; represents differences between adjacent normal skin and lgCMN tissues in T-test. Error bars: mean ± SD. lgCMN, large to giant congenital melanocytic nevi; NS, no significance; T, tumor; N, normal; SD, standard deviation.
